# Providing Accessible ReCreation Outdoors–User-Driven Research on Standards: Protocol for Mobile and Web-Based Interviews for Winter Assessments

**DOI:** 10.2196/38715

**Published:** 2022-10-07

**Authors:** Mike Prescott, Stéphanie Gamache, W Ben Mortenson, Krista L Best, Marie Grandisson, Mir Abolfazl Mostafavi, Delphine Labbé, Ernesto Morales, Atiya Mahmood, Jaimie Borisoff, Bonita Sawatzky, William C Miller, Laura Yvonne Bulk, Julie M Robillard, François Routhier

**Affiliations:** 1 Center for Interdisciplinary Research in Rehabilitation and Social Integration Centre Intégré Universitaire de Santé et de Services Sociaux de la Capitale-Nationale Québec, QC Canada; 2 Centre for Research in Geospatial Data and Intelligence Université Laval Québec, QC Canada; 3 Department of Occupational Science & Occupational Therapy University of British Columbia Vancouver, BC Canada; 4 International Collaboration on Repair Discoveries Vancouver, BC Canada; 5 GF Strong Rehabilitation Research Program Vancouver, BC Canada; 6 Department of Rehabilitation Université Laval Québec, QC Canada; 7 Department of Geomatics Sciences Université Laval Québec, QC Canada; 8 Disability and Human Development Department University of Illinois Chicago, IL United States; 9 Department of Gerontology Simon Fraser University Vancouver, BC Canada; 10 Rehabilitation Engineering Design Lab British Columbia Institute of Technology Burnaby, BC Canada; 11 Department of Orthopedics University of British Columbia Vancouver, BC Canada; 12 Department of Medicine Division of Neurology University of British Columbia Vancouver, BC Canada

**Keywords:** parks, accessibility, standards, user-oriented research, winter, disability, access, participatory, national parks, barriers, participation, Canada, national park, participation, outdoor, activity, standard, interview, safe, virtual, summer, data, mix-method, development

## Abstract

**Background:**

Although there have been recent efforts to improve access to Canadian national parks, many remain not fully accessible to people with disabilities. Winter conditions, in particular, present challenges that limit their participation in outdoor activities.

**Objective:**

This study aimed to develop a novel method to assess park access during winter, which will inform recommendations for national park standards to meet the needs of all park visitors (regardless of ability) during winter conditions.

**Methods:**

A larger participatory mixed methods research project exploring park access was adapted. A 3-phase approach has already been proposed to achieve the study objectives. In the first phase, a scoping review of the existing accessibility standards will be conducted. In the second phase, objective audits of trails and features in 6 parks, 3 in western Canada and 3 in eastern Canada, will be conducted, as well as mobile interviews with 24 various participants in each region regarding their experiences of and recommendations for improving the park’s accessibility. In the final phase, a Delphi participatory consensus development process will be used, based on the data gathered in the first 2 phases, to prioritize recommendations for standards. This paper will focus on the second phase of the study, specifically on whether the in-person winter mobile interviews (ie, walking and wheeling interviews) with people who have a wide range of disabilities while visiting 3 parks in 2 provinces were modified. Changes were made to accommodate the extreme winter weather conditions in Quebec while using safe and informative data collection methods.

**Results:**

In Quebec, one park, where winter conditions are safer, has been assessed in person (n=4). Web-based interviews were used to facilitate the assessment of other winter and summer conditions in two other parks (n=8). Winter and web-based interviews were completed in April 2022. Data are currently being collected and analyzed, and results will be completed by December 2022.

**Conclusions:**

We expect that adapting the protocol to gather further information on winter conditions and access to parks will provide high-quality and rich data to better inform park access standards. This participatory mixed methods research will inform the development of park standards that consider the accessibility needs of all people.

**International Registered Report Identifier (IRRID):**

DERR1-10.2196/38715

## Introduction

### Background

The purpose of this study is to inform accessibility standards in Canadian national parks. Such technical standards do not exist. The protocol, which was previously published [1], reported a participatory approach that will be used with people with disabilities in British Columbia (BC) and Quebec, Canada. A 3-phase approach has already been proposed to achieve the study objectives. In the first phase, a scoping review of the existing accessibility standards will be conducted. In the second phase, objective audits of trails and features in 6 parks, 3 in western Canada and 3 in eastern Canada, will be conducted, as well as mobile interviews with 24 various participants in each region regarding their experiences of and recommendations for improving the park’s accessibility. In the final phase, a Delphi participatory consensus development process will be used, based on the data gathered in the first 2 phases, to prioritize recommendations for standards.

This paper describes the modifications made to address specific challenges related to extreme winter conditions that were experienced at the beginning of the study and, thus, the second phase of the study.

Winter conditions can present serious obstacles for individuals living with a wide range of disabilities (snow and ice made walking dangerous, tires and casters becoming stuck in the snow, difficulty ascending inclines or ramps, and cold hands while using controls or pushing rims, frozen batteries, seat cushions or backrests, or electronics) [2-6]. Having initiated our research activities, we have identified some challenges in carrying out interviews with certain disability groups during winter weather conditions. For example, in discussions with walker and cane users, we learned that the distances and unsafe trail conditions during winter were key barriers to completing the in-person mobile interviews as originally proposed. Thus, our data collection will be incomplete if we rely solely on in-person interviews at the park. We found that even moderate to easy park conditions can be too challenging for some participants, in all weather conditions. As a result, the distances participants can travel limits the number of features they are able to assess and provide feedback on. This is intensified in cold winter conditions where the health risks to participants and the reduction in distances they are able to travel will significantly reduce the breadth and depth of data we are able to collect.

### Objectives

The overall goals of the main study remain the following: (1) to identify park accessibility standards that exist internationally, (2) to identify the accessibility challenges that people with disabilities face in park environments, and (3) to prioritize and recommend accessibility standards for national parks.

The specific objective of this paper is to describe the modified protocol that will be used to inform park standards in summer and winter conditions in Quebec. These protocol modifications will only be made in Quebec, where winter conditions pose more difficult or dangerous experimental conditions, including the transportation risks owing to driving to the mountain parks at this time of the year.

Advisory committees were created in both provinces (Quebec and BC), including individuals with a variety of disabilities (one in each province) to ensure the consideration of inputs or concerns of these individuals in the research project through a participatory research approach. These committees include individuals with mobility, visual, and hearing disabilities; intellectual disabilities; autism spectrum disorder; dementia; and Alzheimer disease. Quebec’s committee was specifically solicited to validate the proposed modifications to the protocol described in this paper.

## Methods

### Overview

Modifications have been made to the second phase of the project that involves in-person mobile interviews. The proposed mobile interview protocol was previously published [1]. Interviews will take place in the park assigned to the participants. The interviews will be administered by trained researchers. The mobile interview will take approximately 2 hours along 3 predetermined routes of 500-1300–m length during both summer and winter.

In Quebec, 2 of 3 parks will be evaluated through web-based interviews in winter (n=8 participants). One park, located in an urban location with nearby amenities, will be evaluated using the initial in-person mobile interview protocol format (n=4 participants), as participant safety can be assured. Web-based interviews facilitate the collection of feedback on features that were planned and, beyond this, by including footage of park elements that are not in the parks, which we selected to be assessed in person. This approach will allow us to collect data when we otherwise might not be able to because most mobility-aid users will be unable to get to or use the trails in winter.

Web-based interviews will also be used to explore access issues in summer conditions with participants who will take part in web-based interviews to allow for comparison. This will ensure comparable results, help inform national standards more effectively, retain methodological consistency, and enable us to gather data from people who would not be able to participate in our original protocol. These adaptations are an example of the participatory nature of the study where the concerns of participants were considered to refine the mechanisms for data collection.

### Mobile Versus Web-Based Interviews

In Fall 2021, the weather became very cold with snow accumulation in Quebec. Four in-person mobile interviews were conducted as originally planned, at which time we observed that participants provided fewer details when answering questions during the in-person winter mobile interviews because they felt uncomfortable (eg, too cold). To address this challenge, we attempted to reduce burden on participants by focusing on questions about features that were altered owing to weather.

The data collection plan retains the preinterview survey from the original protocol and adapts the in-person mobile interview to take into consideration participant burden [1]. Participants who take part in the in-person winter mobile interviews will also complete a summer in-person mobile interview. This strategy will allow us to collect rich data in both winter and summer conditions, while ensuring the comfort and safety of the participants. Table 1 presents a summary of each step.

In addition to the in-person mobile interviews, we will perform web-based interviews. Videos and pictures of trails and features that are similar to those found in national parks were collected from parks in the Quebec City region (eg, Parc Jacques Cartier, Forêt Montmorency, and Plaines of Abraham) both in summer and winter as well as web-based images from national parks to depict the breadth of potential activities available in parks across seasons and the potential for accessibility barriers. Web-based summer interviews will be conducted at the same time for methodological consistency by using the same approach as the web-based winter interviews.

The web-based interviews will not be mobile interviews. The aim is not to comment on a trail as we view it in its entirety but rather to show participants various park features in different contexts to obtain as much feedback as possible on them. The videos and pictures will elicit impressions and opinions to complete a semistructured interview. The web-based interviews will be conducted in the participants’ home or at the research center depending on participant preference. Participants will review videos and pictures of features and trails on a computer monitor or electronic device. Blind and low-vision individuals will not take part in web-based interviews; the in-person mobile interviews allow them to better experience the environment, which could only be described at great lengths to provide sufficient details to truly inform them. A similar set of questions as those in the in-person interviews will be used in the web-based interviews. This will include items from the Stakeholders’ Walkability/Wheelability Audit in Nature (SWAN-PARKS) instrument and open-ended questions to assess trail and feature accessibility and conditions and explore the positive and negative impressions of the experience. Participants will also be asked to provide recommendations for improving the interview experience.

However, way-finding exercises as described in the original protocol, such as estimating distance and slope, pointing to the origin of the route, and sketching maps of the route, which are part of the in-person mobile interviews, will not be conducted during the web-based interviews [1].

**Table 1:** Summary of steps of in-person mobile interviews from the original protocol and modifications for web-based interviews.

Step and summary		Status of modification
**1. Preinterview survey**		
	Web-based questionnaire (Qualtrics) about sociodemographic characteristics (eg, age and sex), disability and mobility status (eg, diagnoses and assistive aids used), subjective wayfinding skills, preferences for park settings and activities, and transport mode to parks	Unchanged, will be done for web-based interviews in the same manner as the in-person mobile interviews
**2. Mobile interviews**		
	Interviews in the park assigned to the participant [7-9] (administered by trained researchers) along 3 predetermined routes of 500-1300–m length (recorded audio and film)	Unchanged for in-person summer and winter mobile in-person interviews
	Map of the intended route of exercise to ask for expectations	In-person summer and winter mobile interviews
	Structured questions (presence or absence of features or characteristics): Stakeholders’ Walkability/Wheelability Audit in Nature (SWAN-PARKS) tool	Only for in-person summer mobile interviews
	Semistructured questions (about their experiences related to way-finding and wayfaring)	In-person summer and winter mobile interviews
**3. Postroute interview questions (for each of the 3 routes)**		
	Objective spatial skills test: orientation and estimation skills. Participants will be positioned at a predefined location and asked to point a compass in the direction of the origin of the route. They will also be asked to estimate the distance and slope to a predefined landmark in the distance [10,11]	Only for in-person summer mobile interviews
	Rate the route on a 7-point Likert scale: perceived physical demand, mental demand, safety, enjoyment, and confidence to find their way independently	In-person summer and winter mobile interviews
	Recall the route verbally or by drawing the route and all its features onto a route map [12]	Only for in-person summer mobile interviews
	Describe the wayfaring and wayfinding experiences overall and provide additional feedback and recommendations	In-person summer and winter mobile interviews. For winter, changes due to seasonality will be noted

### Types of Interviews

Participants of the web-based interviews will complete the data evaluations for both summer and winter conditions using the methods described above. As for the in-person mobile interviews, including those conducted during winter, there are no changes to the protocol followed in BC (the second site), and all interviews will be conducted on site. In Quebec, 4 in-person winter mobile interviews will be conducted at Plains of Abraham (the participants are already recruited), where conditions can be mitigated more easily. The remaining 8 will be web-based interviews.

### Sample Distribution

The number of people to be interviewed and the distribution of participants by disability or mobility type will remain unchanged; that is, a purposive sample of 48 people (24 at each site) with a broad range of disabilities, who use a variety of mobility devices, will be recruited. To be included, participants will need to be at least 18 years of age, able to travel approximately 3 km with rests over a 2-3–hour period, and able to communicate directly with researchers (verbally) or indirectly through an assistant or attendant. Participants will be recruited through partners and participants from previous studies and selective advertising if necessary.

We intend to recruit 24 participants for summer (3 manual wheelchair users, 3 power wheelchair users, 3 scooter users, 3 people who use walkers, 3 people who use canes or crutches, 2 people who are D/deaf and hard of hearing, 3 people who are blind, and 4 people with cognitive impairments) and 12 for winter interviews at each site.

### Participants

Table 2 presents an overview of the participants’ distribution in Quebec. Overall, 24 participants will be recruited in Quebec (8 for Plains of Abraham—4 of whom will participate in both the in-person summer and the winter mobile interviews and 4 will participate in only the summer interviews; 4 in-person summer mobile interviews each for Jacques-Cartier National Park and Forêt Montmorency; and 8 web-based interviews that include both summer and winter conditions).

**Table 2:** Sample distribution (Quebec) according to participants characteristics.

	In-person mobile interview at Plains of Abraham during summer	In-person mobile interview at Plains of Abraham during winter	In-person mobile interview at Jacques-Cartier National Park during summer	In-person mobile interview at Forêt Montmorency during summer	Web-based interview
Wheeled mobility	1 with a scooter, 1 with a power wheelchair (PWC), and 1 with a manual wheelchair (MWC)	1 with a PWC and 1 with an MWC	1 with a scooter and 1 with a PWC	1 with a PWC	1 with a scooter and 2 with MWCs
Walkers, canes, or crutches	1 with a walker and 1 with a cane	None	None	1 with a cane	2 with walkers and 1 with a cane
Visual disability	1	1	1	1	None
Hearing disability	1	None	None	1	None
Cognitive disability	1	1	1	None	2
Total	8	4 of the 8	4	4	8
Total during summer (n=24)	8	None	4	4	8
Total during winter (n=12)	None	4	None	None	8

### Data Analysis: Descriptive Analysis

Transcripts generated from the in-person mobile and web-based interviews will document what was being said or observed and by whom. Pertinent quotes will be coded to reflect the feature or experience being explained (way-finding or wayfaring) by the participant and any observation made by the researchers [1].

For in-person mobile interviews, the quotes and their codes will be digitized in the geographical information system (GIS) at the location that it occurred. This will be linked to the participant survey responses through their ID as a separate file in the GIS (delimited file without spatial information) [1].

For the web-based interviews, as for the in-person mobile interviews, a mixed methods coding process will be used. We identified a list of codes in accordance with the content of the web-based interview guides, and we will adjust the codes in accordance with the emerging data [13]. According to Linneberg and Korsgaard [14], “As the research process develops, so does the type of coding, which also allows the researcher to move from basic descriptive codes toward answering the research question posed.“ Web-based interviews will not be analyzed using the spatial transcript method as described in the original protocol because the activity will not occur in the parks; therefore, there will be no geospatial contextual information available.

### Ethical Considerations

The study was approved by Behaviour Research Ethics Boards at the Centre intégré universitaire de santé et de services sociaux de la Capitale-Nationale (Project #2021-2120) and the Research Ethics Board at the University of British Columbia (H20-04036). Approval was also obtained from the regional health authorities at each site. All study participants will provide informed consent. Evaluation in parks started in August 2021, and web-based interviews started in March 2022.

## Results

Funding for this study was obtained from Accessibility Standards Canada. Using the web-based interviews along with the already proposed in-person mobile interviews allows us to examine features that participants would not be able to comment on because of topography or weather conditions. The results support the development of a spatial transcript and thematic analysis that helps decipher patterns of park experiences between participants across diverse variables such as gender, mobility device use, way-finding abilities, and season. A grounded visualization approach will be used to examine the qualitative and quantitative data derived from the in-person and web-based methods. This involves an iterative analysis of the results, including topographical data derived from open data and the environmental audit such as slope, cross slope, and trail surface conditions to gain a better understanding of the park experience [15,16]. This approach provides a thick, spatially contextualized description of the interactions and perceptions that people with disabilities have with the natural environment and provides the funding agency with more information for the identification of accessibility standards in a park context. Data collection, analysis, and results will be completed by the end of 2022.

## Discussion

### Principal Findings

The purpose of the original protocol previously published [1] was to describe the methodology for informing park accessibility standards. The modified approach proposed in this paper will facilitate data collection on park access for people with diverse disabilities during winter months, as well as the rest of the year, while reducing discomfort and risk. Not everyone has the ability or the capacity to use park installations as they are currently built, regardless of weather conditions. Additionally, cold temperatures, snow accumulation, and icy roads and trails make it difficult to move around parks. As a result, the area that can be assessed in the park would be reduced, and this would limit our ability to collect data. This will also allow us to obtain feedback about features and activities that people with disabilities have never been able to participate in because of accessibility issues. This would assist with site planning (placement of features), which is a significant concern of the Accessibility Standards Canada’s Outdoors Accessibility Committee that is currently developing standards (which author MP is a member).

In addition to allowing us to obtain feedback about more features in the park, web-based interviews may make recruitment more successful. Many of the challenges that limit mobility also affect decisions regarding study participation. Conducting interviews in participants’ homes or at the laboratory will reduce travel demands on participants and mitigate the impact of being outdoors for several hours during the in-person mobile interview.

To our knowledge, this is the first study to leverage a web-based interface for collecting data about outdoor environments with people with disabilities. The potential impacts generated by the modification of the original protocol include the possibility of exploring more barriers and access issues in a wider range of parks and conditions. Most people with disabilities avoid going out in the winter but would still like to be active [4,17]. They might not be aware of the potential opportunities that exist. Using the web-based method allows us to explore these features and better inform accessibility standards. Without the web-based method, this exploration would not be possible.

### Limitations

This project targets national parks. It is hoped that the obtained finding could also be useful in the design of community parks, but these kinds of parks were not specifically targeted in this project. The limitations of this approach are a modest reduction of insights on the real-world experiences of people with disabilities travelling along winter trails and limited feedback about wayfinding requirements. However, these changes are proposed to maximize participant safety, while no adapted equipment is available on site. These limitations are mitigated by the fact that we will complete these activities in the winter in 1 park in Quebec and all 3 parks in BC.

### Conclusions

People with disabilities’ valuable insights on winter conditions and parks will inform accessibility standards to be used in national parks and beyond. Accessibility in winter conditions can be very difficult to attain and very difficult to assess in real-life situations for certain groups. This also applies to certain individuals in summer conditions. By gathering individuals with disabilities’ opinions using a variety of methods that allow individuals to participate in the discussion regarding park access during all seasons while respecting their capacities can provide a solid basis on which to better plan park design to overcome obstacles during all seasons.

## Abbreviations

BC: British Columbia
GIS: geographical information system
SWAN-PARKS: Stakeholders’ Walkability/Wheelability Audit in Nature
